# Supplementary material for: Characterisation and induction of tissue-resident gamma delta T-cells to target hepatocellular carcinoma
Source: Nat Commun. 2022 Mar 16;13:1372. doi: 10.1038/s41467-022-29012-1 (PMC8927126; doi:10.1038/s41467-022-29012-1)
Supplement: Supplementary file 1 — Supplementary Information [file 41467_2022_29012_MOESM1_ESM.pdf]

Characterisation and induction of tissue-resident gamma delta T-cells to target hepatocellular carcinoma

Nekisa Zakeri, Andrew Hall, Leo Swadling, Laura J Pallett, Nathalie M Schmidt, Mariana O Diniz, Stephanie Kucykowicz, Oliver E Amin, Amir Gander, Massimo Pinzani, Brian R Davidson, Alberto Quaglia, Mala K Maini

Supp.Fig.1

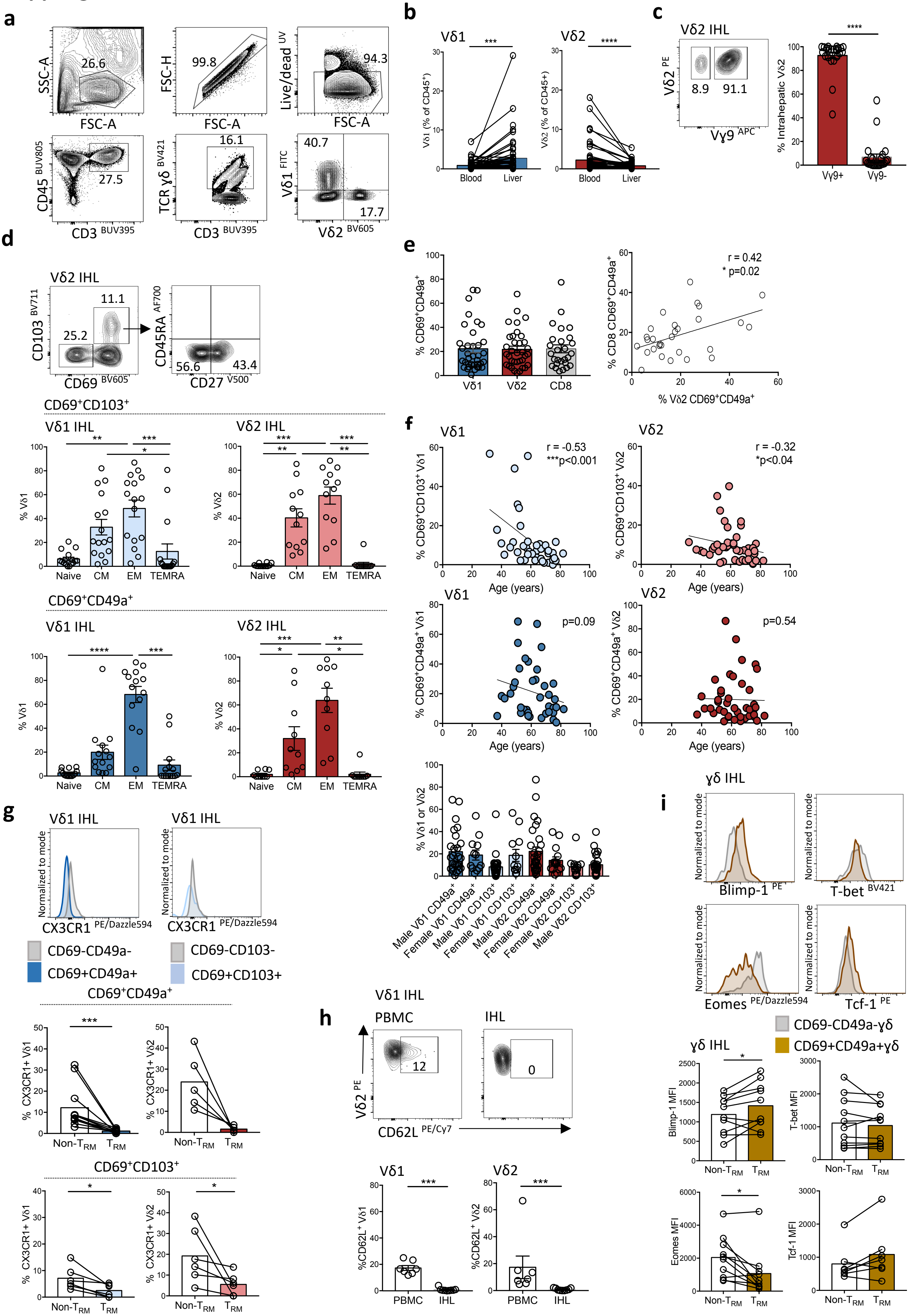

a
SSC-A
FSC-A
26.6
FSC-H
99.8
Live/dead UV
94.3
CD45 BUV805
CD3 BUV395
27.5
TCR γδ BV421
16.1
Vδ1 FITC
Vδ2 BV605
40.7
17.7
b
Vδ1
Vδ2
Vδ1 (% of CD45+)
Vδ2 (% of CD45+)
Blood
Liver
c
Vδ2 IHL
Vδ2 PE
Vγ9 APC
8.9
91.1
% Intrahepatic Vδ2
Vγ9+
Vγ9-
d
Vδ2 IHL
CD103 BV711
CD69 BV605
11.1
25.2
CD45RA AF700
CD27 V500
56.6
43.4
CD69+CD103+
Vδ1 IHL
Vδ2 IHL
% Vδ1
% Vδ2
Naive
CM
EM
TEMRA
CD69+CD49a+
e
% CD69+CD49a+
Vδ1
Vδ2
CD8
r = 0.42
* p=0.02
% CD8 CD69+CD49a+
% Vδ2 CD69+CD49a+
f
r = -0.53
***p<0.001
r = -0.32
*p<0.04
% CD69+CD103+ Vδ1
% CD69+CD103+ Vδ2
Age (years)
p=0.09
p=0.54
% CD69+CD49a+ Vδ1
% CD69+CD49a+ Vδ2
% Vδ1 or Vδ2
Male Vδ1 CD49a+
Female Vδ1 CD49a+
Male Vδ1 CD103+
Female Vδ1 CD103+
Male Vδ2 CD49a+
Female Vδ2 CD49a+
Female Vδ2 CD103+
Male Vδ2 CD103+
g
Vδ1 IHL
Normalized to mode
CX3CR1 PE/Dazzle594
CD69-CD49a-
CD69+CD49a+
CD69-CD103-
CD69+CD103+
CD69+CD49a+
% CX3CR1+ Vδ1
% CX3CR1+ Vδ2
Non-TRM
TRM
CD69+CD103+
h
Vδ1 IHL
PBMC
IHL
Vδ2 PE
CD62L PE/Cy7
12
0
%CD62L+ Vδ1
%CD62L+ Vδ2
i
γδ IHL
Blimp-1 PE
T-bet BV421
Eomes PE/Dazzle594
Tcf-1 PE
CD69-CD49a-γδ
CD69+CD49a+γδ
Blimp-1 MFI
T-bet MFI
Eomes MFI
Tcf-1 MFI

Supplementary Figure 1. Compartmentalisation of Vδ1 and Vδ2 T-cells with a tissue-resident phenotype in human liver. a, Representative flow cytometry plot of the gating strategy for γδ T-cells and subsets (sequential gating on lymphocytes, single cells, live cells, $CD45^{+}CD3^{+}$ T-cells, γδ T-cells, Vδ1 and Vδ2 T-cells). **b,** Frequency of Vδ1 and Vδ2 T-cells in paired blood and tumour-free liver tissue samples, expressed as a percentage of total $CD45^{+}$ leukocytes (n=56; Vδ1 p=0.0008; Vδ2 p<0.0001). **c,** Frequency of intrahepatic $V\gamma9^{-}$ versus $V\gamma9^{+}$ Vδ2 T-cells (n=24; p<0.0001). **d,** Memory phenotype (naïve $CD27^{+}CD45RA^{+}$, central memory $CD27^{+}CD45RA^{-}$, effector memory $CD27^{-}CD45RA^{-}$, terminally differentiated effector memory (T_{EMRA}) $CD27^{-}CD45RA^{+}$) of $CD69^{+}CD103^{+}$ or $CD69^{+}CD49a^{+}$ intrahepatic Vδ1 and Vγ9Vδ2 T_{RM} (n=10-16; Vδ1 $CD69^{+}CD103^{+}$ p=0.0098, p=0.0004, p=0.02; Vδ1 $CD69^{+}CD49a^{+}$ p<0.0001, p=0.0008; Vγ9Vδ2 $CD69^{+}CD103^{+}$ p=0.007, p=0.0003, p=0.007, p=0.0003; Vγ9Vδ2 $CD69^{+}CD49a^{+}$ p=0.01, p=0.0008, p=0.02, p=0.001). **e**, Summary data of $CD69^{+}CD49a^{+}$ expression on intrahepatic Vδ1, Vγ9Vδ2 and αβCD8 T-cells (n=41); correlation of $CD69^{+}CD49a^{+}$ expression on Vγ9Vδ2 and $\alpha\beta CD8^{+}$ T-cells. **f,** Correlation of levels of $CD69^{+}CD103^{+}$ and $CD69^{+}CD49a^{+}$ expression on Vδ1, Vγ9Vδ2 T-cells with patient age and comparison with patient sex (n=38). **g,** CX3CR1 expression by intrahepatic Vδ1 and Vδ2 T_{RM} ($CD69^{+}CD49a^{+}$ or $CD69^{+}CD103^{+}$) (n=5-11; Vδ1 p=0.001, p=0.03; Vγ9Vδ2 p=0.03). **h,** CD62L expression by Vδ1 and Vδ2 T-cells within PBMCs compared to IHL (n=10; Vδ1 p=0.0002; Vγ9Vδ2 p=0.0002). **i,** Intranuclear expression of Blimp-1 (p=0.048), T-bet, Eomes (p=0.01), Tcf-1 transcription factors (median fluorescence intensity) by intrahepatic $CD69^{+}CD49a^{+}$ $\gamma\delta T_{RM}$ in comparison to $CD69^{-}CD49a^{-}$ γδ T-cells (n=10). Each symbol represents a study participant, with bars showing the mean ± SEM. Two-tailed p-values were determined by Wilcoxon paired test **(b-c,g,i)**, Spearman rank correlation test **(e,f)**, Mann-Whitney test **(h)**, or Friedman test with Dunn's post-hoc test for multiple comparisons **(d,e,f)**. * p<0.05; ** p<0.01; *** p<0.001; ****p<0.0001.

Supp.Fig.2

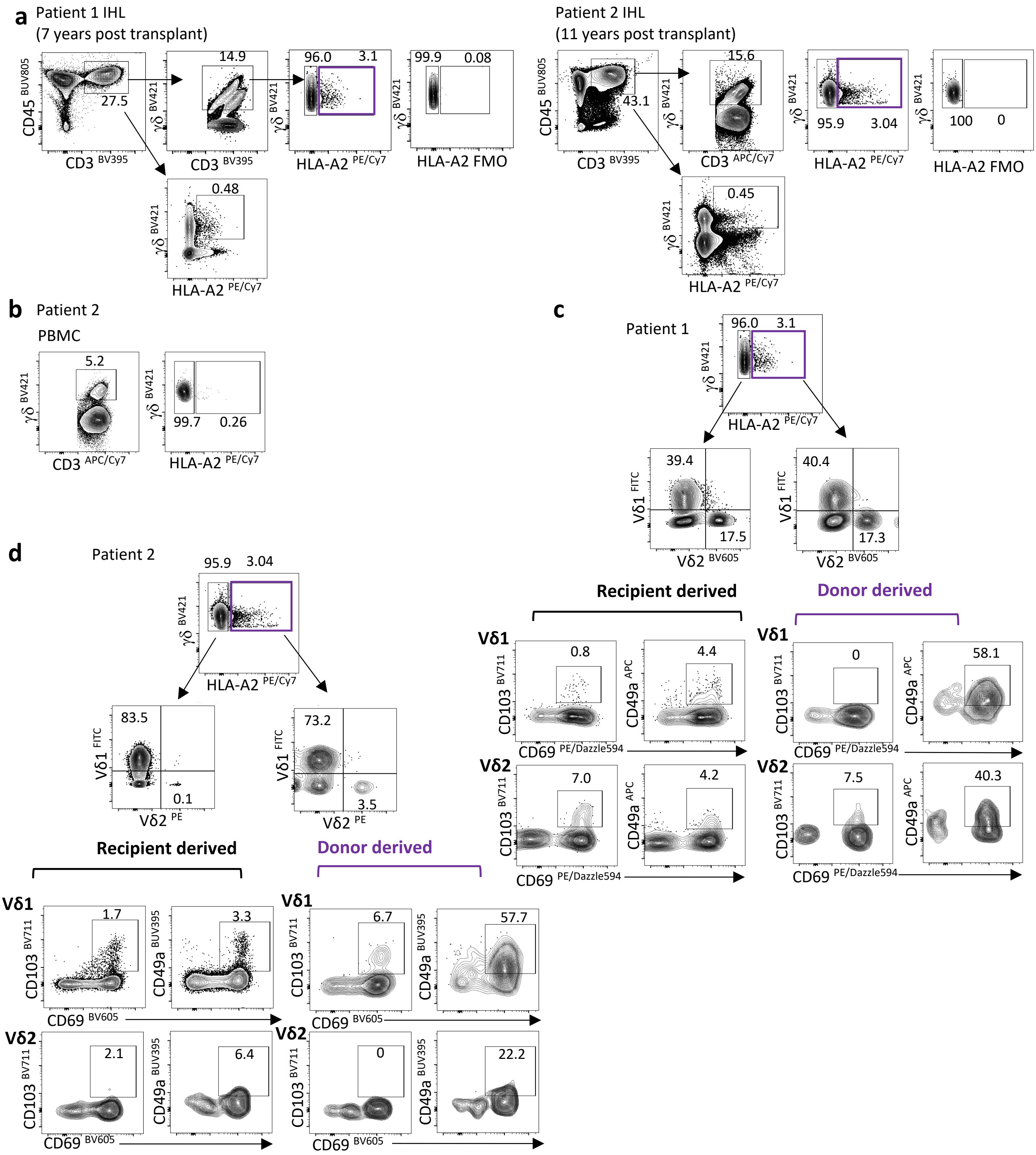

Supplementary Figure 2. Long-lived hepatic retention and replenishment of Vδ1 and Vɣ9Vδ2-T$_{RM}$. a, Flow cytometry gating for the identification of donor (HLA A2$^+$) and recipient (HLA A2$^-$) derived γδ T-cells from two explants obtained 7 years or 11 years following HLA-mismatched liver transplantation, using HLA-A2 monoclonal antibody fluorescence minus one (FMO) controls. **b,** Flow cytometry plot demonstrating absence of donor-derived (HLA A2$^+$) γδ T-cells in PBMCs from the patient who received an HLA-mismatched liver transplant 11-years prior. **c,d,** Flow cytometry plot of CD69$^+$CD103$^+$ or CD69$^+$CD49a$^+$ expression on donor-derived (HLA A2$^+$) and recipient-derived (HLA A2$^-$) intrahepatic Vδ1 and Vɣ9Vδ2 T-cell subsets from an explant obtained 11 years **(c)** and 7 years **(d)** following the original HLA-mismatched liver transplantation.

Supp.Fig.3

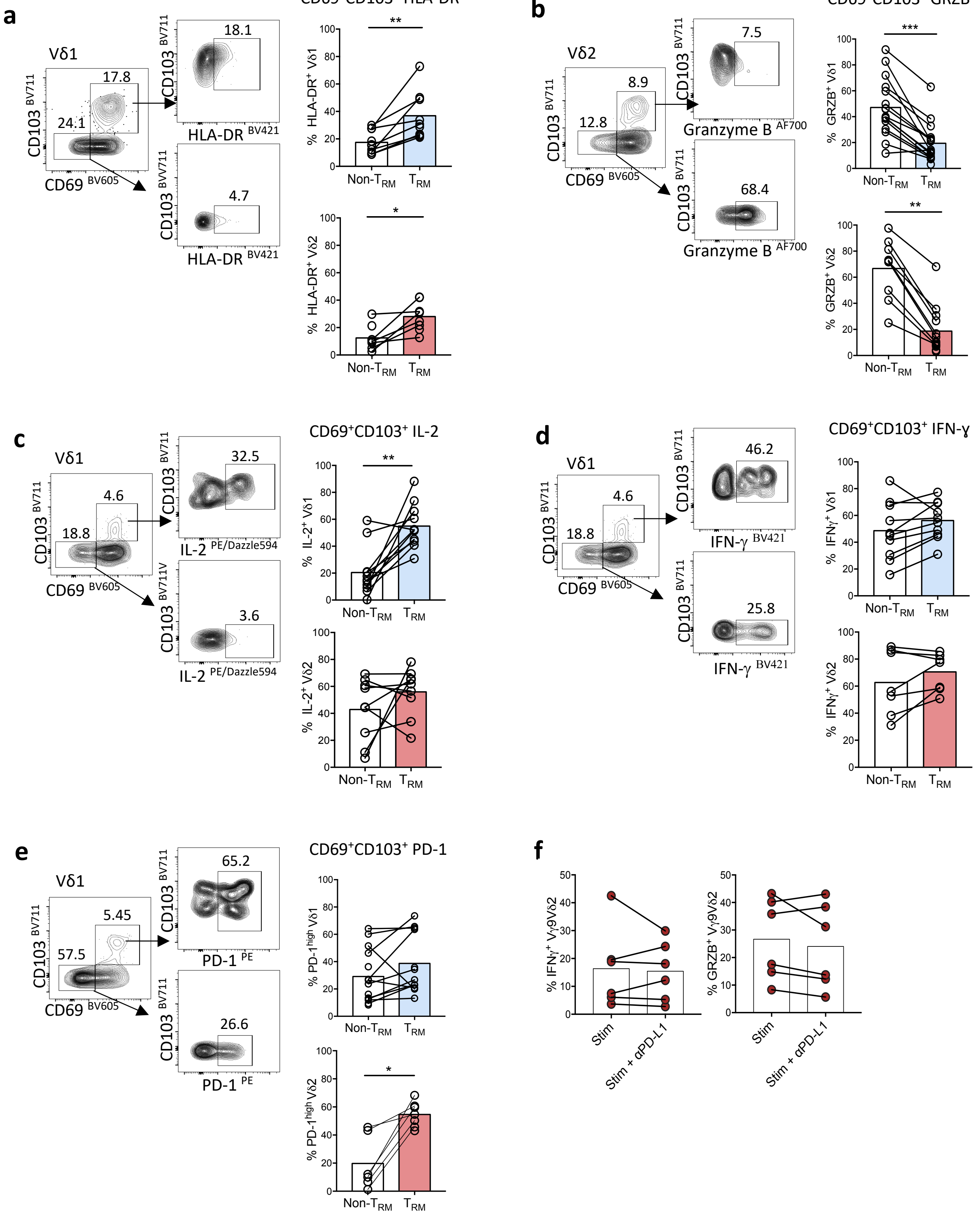
a
Vδ1
17.8
24.1
CD103 BV711
CD69 BV605
18.1
4.7
HLA-DR BV421
CD69+CD103+ HLA-DR
% HLA-DR+ Vδ1
% HLA-DR+ Vδ2
**
*
Non-TRM
TRM
b
Vδ2
8.9
12.8
7.5
68.4
Granzyme B AF700
CD69+CD103+ GRZB
% GRZB+ Vδ1
% GRZB+ Vδ2
***
**
c
Vδ1
4.6
18.8
32.5
3.6
IL-2 PE/Dazzle594
CD69+CD103+ IL-2
% IL-2+ Vδ1
% IL-2+ Vδ2
d
Vδ1
46.2
25.8
IFN-γ BV421
CD69+CD103+ IFN-γ
% IFNγ+ Vδ1
% IFNγ+ Vδ2
e
Vδ1
5.45
57.5
65.2
26.6
PD-1 PE
CD69+CD103+ PD-1
% PD-1high Vδ1
% PD-1high Vδ2
f
% IFNγ+ Vγ9Vδ2
% GRZB+ Vγ9Vδ2
Stim
Stim + αPD-L1

Supplementary Figure 3. Distinct functional profile of hepatic $\gamma\delta T_{RM}$. (a-e) Representative flow cytometry plots and summary data of *ex vivo* functional profile of intrahepatic $CD69^{+}CD103^{+}$ $V\delta1$ and $V\gamma9V\delta2$ T_{RM} compared to non-T_{RM} ($CD69^{-}CD103^{-}$) counterparts. **a,** HLA-DR expression (n=10; p=0.004, p=0.03). **b,** unstimulated Granzyme B expression (n=16; p=0.0001, p=0.004). **c,** IL-2 expression following 4-hour PMA and Ionomycin *ex vivo* stimulation (n=11; p=0.002). **d,** IFN-γ expression after 4-hour PMA and Ionomycin stimulation (n=10). **e,** unstimulated PD-1 expression (n=10; p=0.03). **f,** IFN-γ and Granzyme B expression by intrahepatic $V\gamma9V\delta2$ T-cells after 16-hour TCR-$\gamma\delta$ stimulation with or without addition of anti-PD-L1 blockade (n=6). Each symbol represents a study participant, with bars showing the mean. Two-tailed p-values determined by Wilcoxon matched-pairs signed rank test **(a-f)** * p<0.05; ** p<0.01; *** p<0.001.

Supp.Fig.4

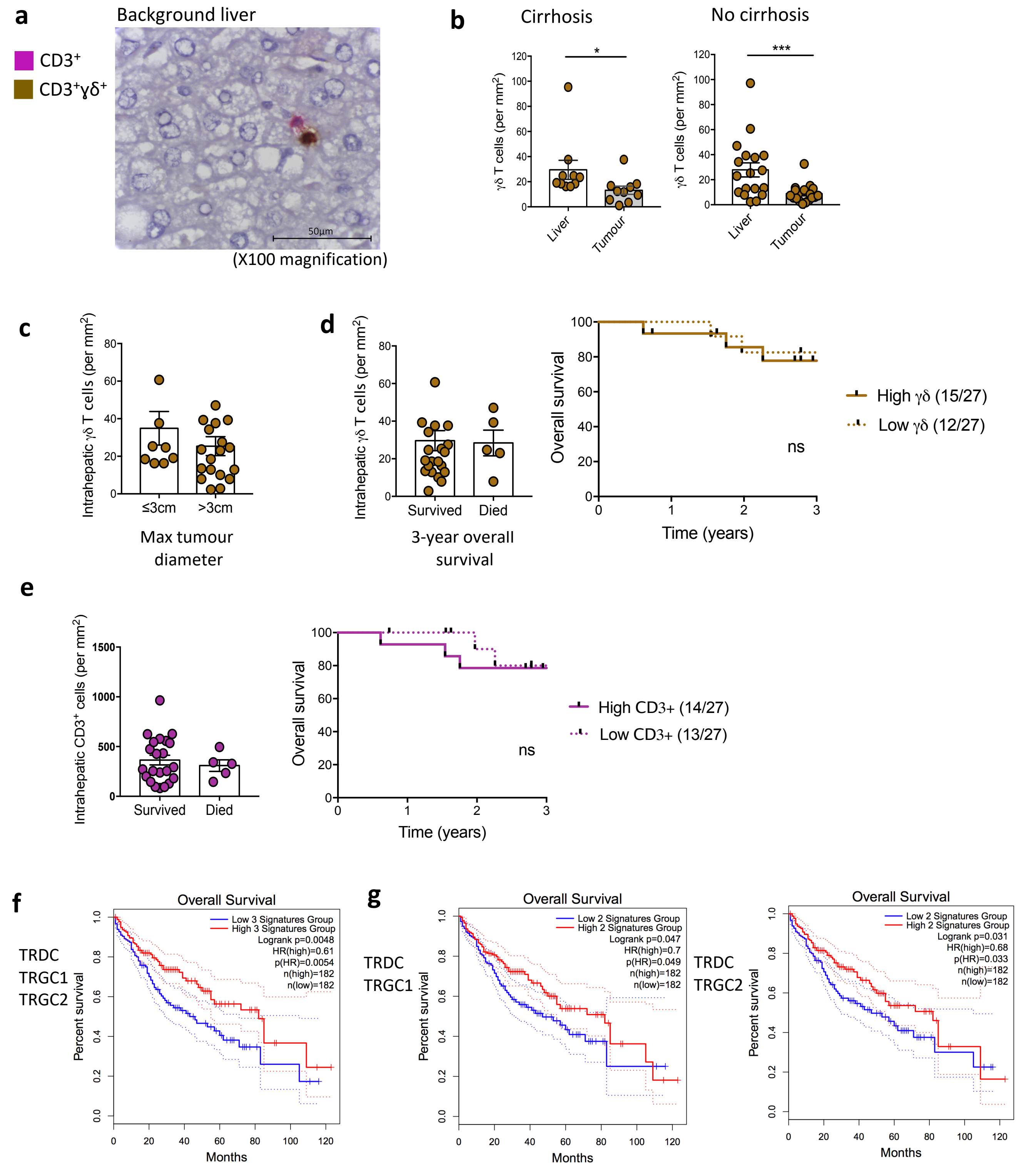
a
Background liver
CD3+
CD3+γδ+
50µm
(X100 magnification)
b
Cirrhosis
No cirrhosis
γδ T cells (per mm2)
*
***
Liver
Tumour
c
Intrahepatic γδ T cells (per mm2)
≤3cm
>3cm
Max tumour diameter
d
Survived
Died
3-year overall survival
Overall survival
Time (years)
High γδ (15/27)
Low γδ (12/27)
ns
e
Intrahepatic CD3+ cells (per mm2)
Survived
Died
Overall survival
Time (years)
High CD3+ (14/27)
Low CD3+ (13/27)
ns
f
TRDC
TRGC1
TRGC2
Overall Survival
Low 3 Signatures Group
High 3 Signatures Group
Logrank p=0.0048
HR(high)=0.61
p(HR)=0.0054
n(high)=182
n(low)=182
Percent survival
Months
g
TRDC
TRGC1
Overall Survival
Low 2 Signatures Group
High 2 Signatures Group
Logrank p=0.047
HR(high)=0.7
p(HR)=0.049
n(high)=182
n(low)=182
Percent survival
Months
TRDC
TRGC2
Overall Survival
Low 2 Signatures Group
High 2 Signatures Group
Logrank p=0.031
HR(high)=0.68
p(HR)=0.033
n(high)=182
n(low)=182
Percent survival
Months

Supplementary Figure 4. γδ T-cell counts in HCC are associated with tumour size and patient survival. Immunohistochemistry staining of paired background liver and tumour tissue obtained from patients with hepatocellular carcinoma (HCC) undergoing surgical resection. Cell counts performed from 5 randomly selected high-power fields (x20 objective magnification) per sample (n=28 paired samples). **a,** Representative high magnification (x100) immunostaining of non-γδ $CD3^+$ (purple) and $γδ^+$ (brown) T-cells in liver tissue. **b,** γδ T-cell counts (per mm^2) in tumour-free liver and paired HCC with or without underlying cirrhosis ($p=0.048$, $p=0.0005$) (n=28). **c,** Intrahepatic γδ T-cell counts (per mm^2) in small HCC tumours with a maximum diameter of ≤3cm compared to HCC tumours >3cm in diameter (n=28). **d,** Intrahepatic γδ T-cell counts (per mm^2) according to 3-year patient overall survival outcomes (survival data available in n=27; 22/27 survived, 5/27 died); Kaplan Meier graph of overall survival (years post resection) split on the median intrahepatic γδ T-cell count from 27 liver samples. **e,** Intrahepatic non-γδ $CD3^+$ T-cell counts (per mm^2) according to 3-year patient overall survival outcomes. Kaplan Meier graph of overall survival (years post resection) split on the median intrahepatic $CD3^+$ T-cell count from 27 liver samples. **f,** Analysis of γδ-TCR gene signature (TRDC, TRGC1, TRGC2) with overall survival in HCC patients in the Cancer Genome Atlas database (n=364) using GEPIA2 web server. **g,** Analysis of Vγ9Vδ2 TCR (TRDC, TRGC1) and non-Vγ9Vδ2 γδ TCR (TRDC, TRGC2) gene signatures with overall survival in HCC patients (n=364) using the GEPIA2 web server. Two-tailed p-values determined by Wilcoxon paired test **(b)**, Mann Whitney test **(c-e)** Kaplan Meier graphs with Log-rank test **(d-g).** Error bars represent mean ± SEM. ns, not significant; * $p<0.05$; ** $p<0.01$; *** $p<0.001$.

Supp.Fig.5

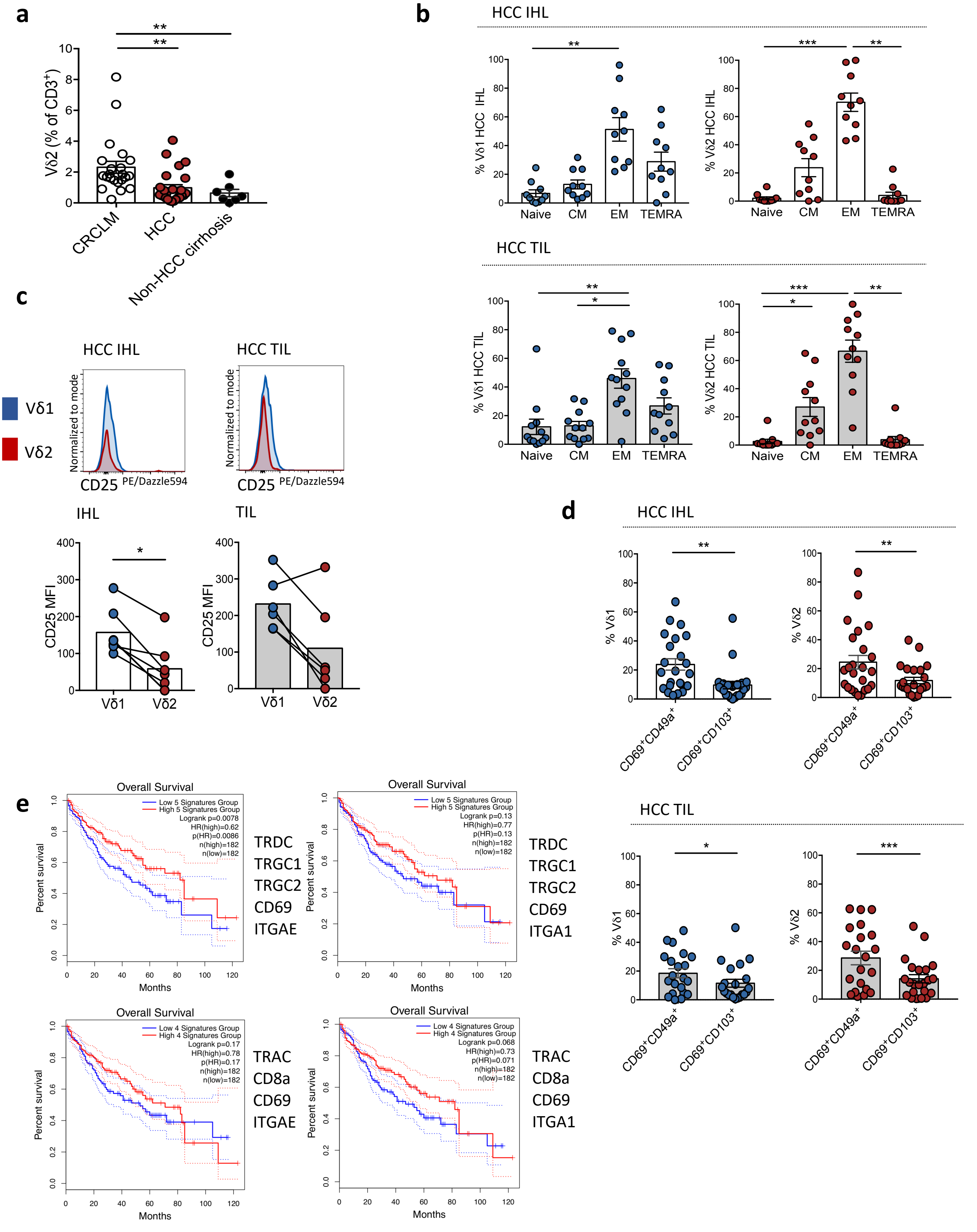
a
Vδ2 (% of CD3+)
CRCLM
HCC
Non-HCC cirrhosis
b
HCC IHL
% Vδ1 HCC IHL
% Vδ2 HCC IHL
Naive
CM
EM
TEMRA
HCC TIL
% Vδ1 HCC TIL
% Vδ2 HCC TIL
c
HCC IHL
HCC TIL
Vδ1
Vδ2
Normalized to mode
CD25 PE/Dazzle594
IHL
TIL
CD25 MFI
d
HCC IHL
% Vδ1
% Vδ2
CD69+CD49a+
CD69+CD103+
HCC TIL
e
Overall Survival
Low 5 Signatures Group
High 5 Signatures Group
Logrank p=0.0078
HR(high)=0.62
p(HR)=0.0086
n(high)=182
n(low)=182
Percent survival
Months
TRDC
TRGC1
TRGC2
CD69
ITGAE
Overall Survival
Low 5 Signatures Group
High 5 Signatures Group
Logrank p=0.13
HR(high)=0.77
p(HR)=0.13
n(high)=182
n(low)=182
TRDC
TRGC1
TRGC2
CD69
ITGA1
Overall Survival
Low 4 Signatures Group
High 4 Signatures Group
Logrank p=0.17
HR(high)=0.78
p(HR)=0.17
n(high)=182
n(low)=182
TRAC
CD8a
CD69
ITGAE
Overall Survival
Low 4 Signatures Group
High 4 Signatures Group
Logrank p=0.068
HR(high)=0.73
p(HR)=0.071
n(high)=182
n(low)=182
TRAC
CD8a
CD69
ITGA1

Supplementary Figure 5. Vγ9Vδ2 T-cells are selectively depleted, but can acquire tissue-residence, within HCC TILs. a, Summary data of Vγ9Vδ2 T-cell frequency in intrahepatic lymphocytes (IHL) isolated from tumour-free liver tissue of CRCLM (n=29), HCC (n=23) or non-HCC cirrhotic livers (n=6), p=0.007, p=0.001. **b,** Memory phenotype (naïve $CD27^+CD45RA^+$, central memory $CD27^+CD45RA^-$, effector memory $CD27^-CD45RA^-$, terminally differentiated effector memory (T_{EMRA}) $CD27^-CD45RA^+$) of Vδ1 and Vγ9Vδ2 T-cells in paired IHL (top) and HCC tumour-infiltrating lymphocytes (TIL) (bottom) (n=11; Vδ1 IHL p=0.002; Vγ9Vδ2 IHL p=0.0004, p=0.002; Vδ1 HCC TIL p=0.001, p=0.01; Vγ9Vδ2 HCC TIL p=0.0004, p=0.001, p=0.049). **c,** CD25 expression (median fluorescence intensity) by Vδ1 and Vγ9Vδ2 T-cells in IHL (n=7; p=0.02) and HCC TIL (n=6)**. d,** Summary data comparison of $CD69^+CD103^+$ and $CD69^+CD49a^+$ expression on Vδ1 and Vγ9Vδ2 T-cells in IHL and HCC TIL (n=25; Vδ1 IHL p=0.002; Vγ9Vδ2 IHL p=0.008; Vδ1 TIL p=0.02; Vγ9Vδ2 TIL p=0.0002). **e,** Analysis of combinations of γδ-TCR (top) or CD8αβ-TCR (bottom) and $CD69^+CD103^+$ (left) or $CD69^+CD49a^+$ (right) gene signatures with overall survival in HCC in the Cancer Genome Atlas database (n=364) using GEPIA2 web server. Two-tailed p-values determined by Kruskal Wallis test with Dunn's post hoc test **(a)**, Friedman test with Dunn's post-hoc test **(b)**, Wilcoxon matched-pairs signed rank test **(c,d),** Kaplan Meier graphs with Log-rank test **(e)**. Error bars, mean ± SEM. * p<0.05; ** p<0.01; *** p<0.001.

Supp.Fig.6

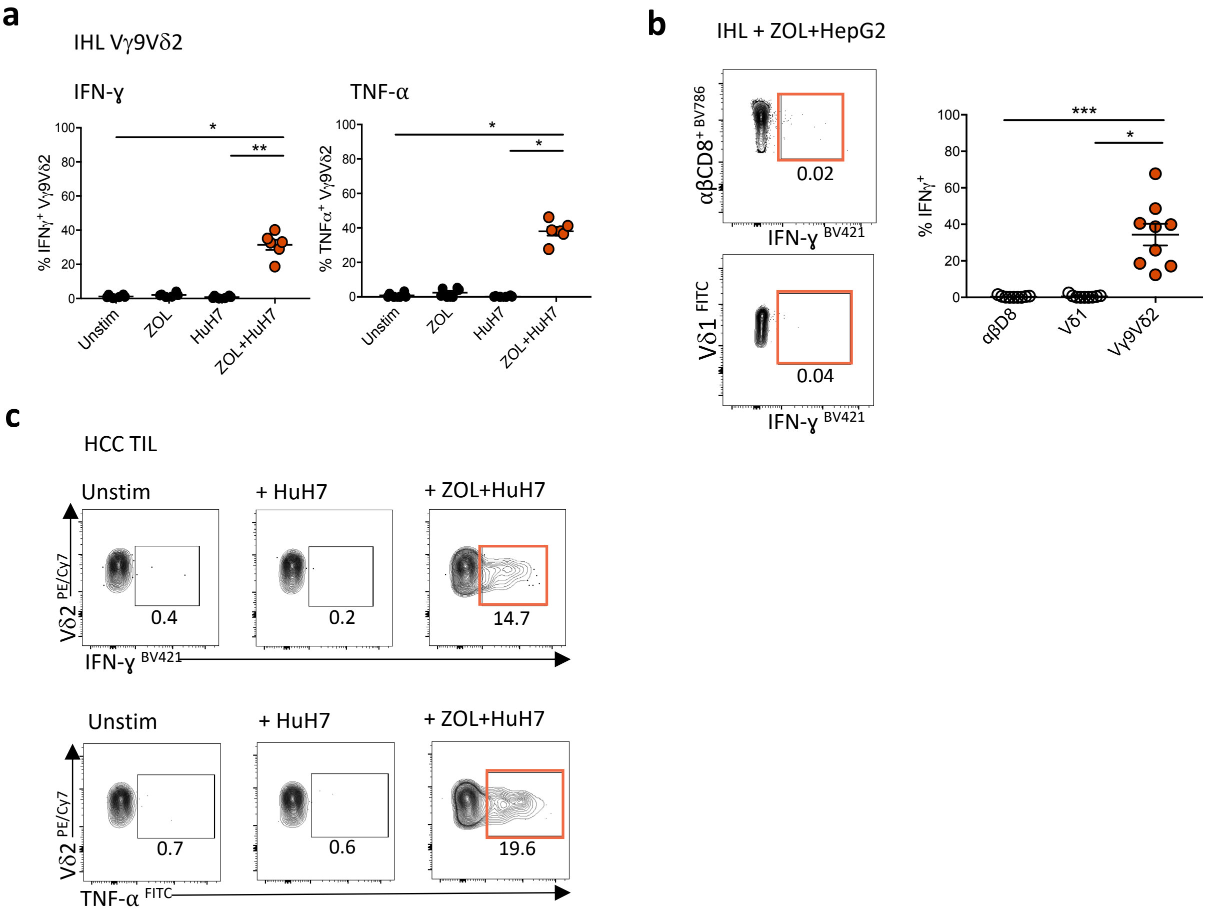

Supplementary Figure 6. Anti-tumour potential of Vγ9Vδ2 T_{RM} against ZOL-sensitised HCC cell lines. a, Summary data of IFN-γ and TNF-α expression by Vγ9Vδ2 T-cells in intrahepatic lymphocytes (IHL) (n=6) either: unstimulated, directly treated with 5μM Zoledronic acid (ZOL), or after co-culture with untreated or ZOL pre-treated HuH7 cells (E:T 2:1 ratio; 0.6 x10^6 IHL to 0.3 x10^6 HuH7 cells, 6 hours co-culture, all conditions performed in duplicate or triplicate); IFN-γ p=0.02, p=0.003; TNF-α p=0.01, p=0.02. **b,** Representative flow cytometry plot and summary data demonstrating selective upregulation of IFN-γ by intrahepatic Vγ9Vδ2 T-cells and no IFN-γ expression by Vδ1 and αβCD8 T-cells after co-culture with ZOL pre-treated HepG2 cells (n=9; p=0.0005, p=0.03). **c,** Flow cytometry plot of IFNγ and TNFα expression by Vγ9Vδ2 T-cells in HCC tumour-infiltrating lymphocytes (TIL) unstimulated, or after co-culture with untreated or ZOL pre-treated HuH7 cells. Two-tailed p-values determined by Friedman test with Dunn's post-hoc test for multiple comparisons **(a,b)**. Error bars represent mean ± SEM. * p<0.05; ** p<0.01; *** p<0.001.

Supp.Fig.7

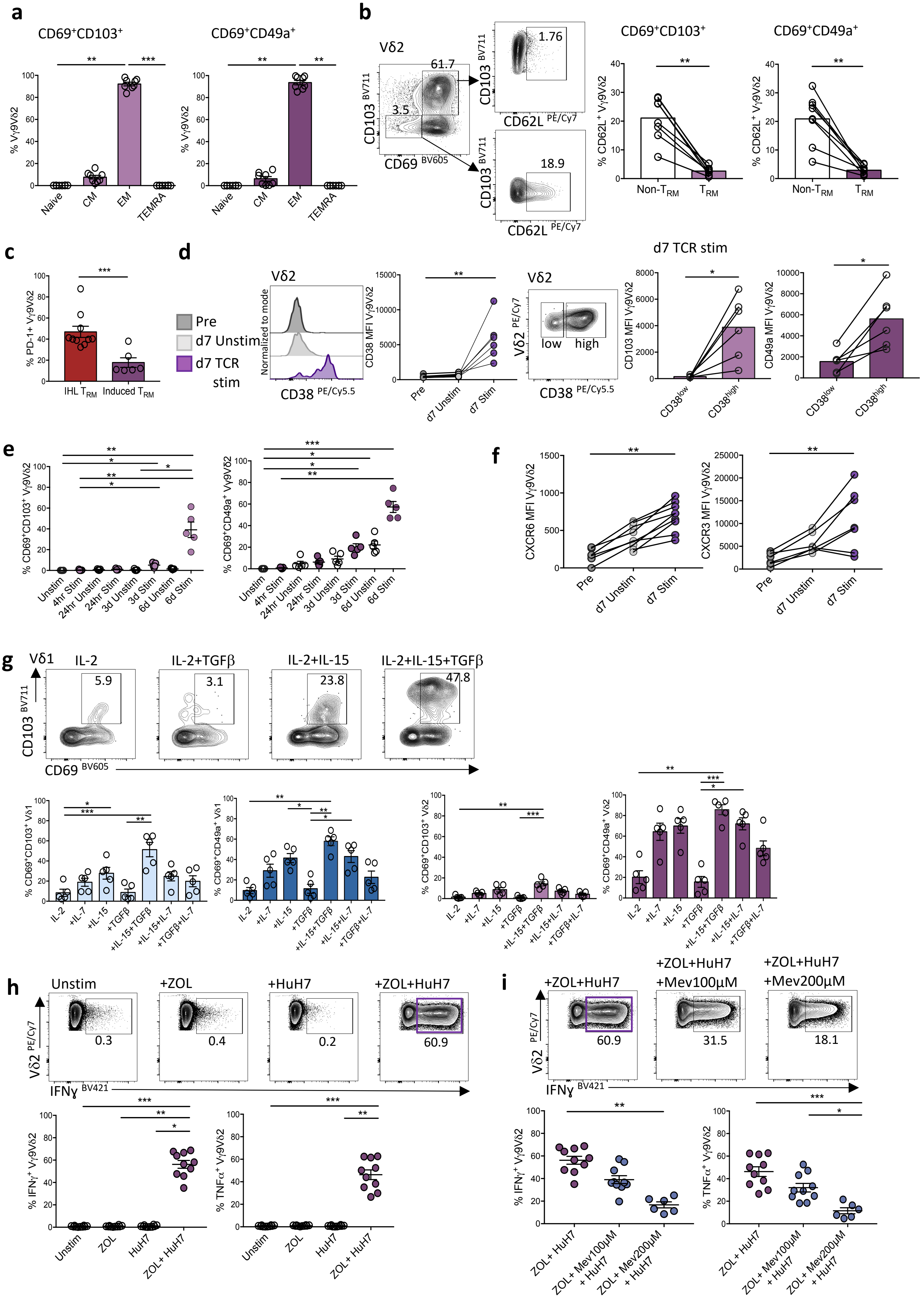
a
CD69+CD103+
CD69+CD49a+
% Vγ9Vδ2
Naive
CM
EM
TEMRA
b
Vδ2
CD103 BV711
CD69 BV605
61.7
3.5
1.76
18.9
CD62L PE/Cy7
% CD62L+ Vγ9Vδ2
Non-TRM
TRM
c
% PD-1+ Vγ9Vδ2
IHL TRM
Induced TRM
d
Pre
d7 Unstim
d7 TCR stim
Normalized to mode
CD38 PE/Cy5.5
CD38 MFI Vγ9Vδ2
d7 Stim
Vδ2 PE/Cy7
low
high
d7 TCR stim
CD103 MFI Vγ9Vδ2
CD49a MFI Vγ9Vδ2
CD38low
CD38high
e
% CD69+CD103+ Vγ9Vδ2
% CD69+CD49a+ Vγ9Vδ2
Unstim
4hr Stim
24hr Unstim
24hr Stim
3d Unstim
3d Stim
6d Unstim
6d Stim
f
CXCR6 MFI Vγ9Vδ2
CXCR3 MFI Vγ9Vδ2
g
Vδ1
IL-2
IL-2+TGFβ
IL-2+IL-15
IL-2+IL-15+TGFβ
5.9
3.1
23.8
47.8
CD103 BV711
CD69 BV605
% CD69+CD103+ Vδ1
% CD69+CD49a+ Vδ1
% CD69+CD103+ Vδ2
% CD69+CD49a+ Vδ2
+IL-7
+IL-15
+TGFβ
+IL-15+TGFβ
+IL-15+IL-7
+TGFβ+IL-7
h
Unstim
+ZOL
+HuH7
+ZOL+HuH7
0.3
0.4
0.2
60.9
Vδ2 PE/Cy7
IFNγ BV421
% IFNγ+ Vγ9Vδ2
% TNFα+ Vγ9Vδ2
ZOL
HuH7
ZOL+ HuH7
i
+ZOL+HuH7
+ZOL+HuH7 +Mev100μM
+ZOL+HuH7 +Mev200μM
60.9
31.5
18.1
ZOL+ Mev100μM + HuH7
ZOL+ Mev200μM + HuH7

Supplementary Figure 7. ZOL expands *de novo* Vγ9Vδ2 T_{RM} from blood, targeting ZOL-sensitised HCC cell lines. a, Memory phenotype (naïve $CD27^+CD45RA^+$, central memory $CD27^+CD45RA^-$, effector memory $CD27^-CD45RA$-, terminally differentiated effector memory (T_{EMRA}) $CD27^-CD45RA^+$) of *de novo* induced blood $CD69^+CD103^+$ (p=0.001, p=0.0003) or $CD69^+CD49a^+$ Vγ9Vδ2 T_{RM} (p=0.001, p=0.0003) after Zoledronic acid (ZOL)/IL-2-based expansion (n=9). **b,** CD62L expression by $CD69^+CD103^+$ or $CD69^+CD49a^+$ expanded blood Vγ9Vδ2 T_{RM} compared to their non-T_{RM} ($CD69^-CD103^-CD49a^-$) Vγ9Vδ2 T-cell counterparts (n=8; p=0.008, p=0.008). **c,** Programmed cell death protein-1 **(**PD-1) expression on ZOL/IL-2 expanded $CD69^+CD49a^+$ Vγ9Vδ2 T_{RM} (n=6) compared to Vγ9Vδ2 T_{RM} directly isolated from liver tissue (n=10; p=0.001). **d,** CD38 expression on Vγ9Vδ2 T-cells in PBMCs after culture with IL-2 +/- TCR-$\gamma\delta$ stimulation using a plated anti-TCR-$\gamma\delta$ antibody (p=0.002); CD103 and CD49a median fluorescence intensity (MFI) on $CD38^{high}$ versus $CD38^{low}$ expressing Vγ9Vδ2 T-cells in PBMCs after 7-days IL-2 and plated TCR-$\gamma\delta$ stimulation (n=6; p=0.03, p=0.03). **e,** $CD69^+CD103^+$ or $CD69^+CD49a^+$ expression on Vγ9Vδ2 T-cells in PBMCs after culture with IL-2 +/- plated TCR-$\gamma\delta$ stimulation at different time points (unstim, 4-hours, 24-hours, 3-days, 6-days) (n=5; $CD69^+CD103^+$ p=0.001, p=0.02, p=0.04, p=0.004, p=0.02; $CD69^+CD49a^+$ p=0.0002, p=0.02, p=0.003, p=0.01). **f,** CXCR6 and CXCR3 expression (MFI) on Vγ9Vδ2 T-cells in PBMCs at baseline and after 7-day culture with IL-2 +/- plated TCR-$\gamma\delta$ stimulation (n=6; CXCR6 p=0.002; CXCR3 p=0.0045). **g,** $CD69^+CD103^+$ or $CD69^+CD49a^+$ expression induced on Vδ1 and Vγ9Vδ2 T-cells in PBMCs after 7-day culture with IL-2, IL-7, IL-15 or TGF-β cytokines (n=5; Vδ1 $CD69^+CD103^+$ p=0.04, p=0.0009, p=0.002; Vδ1 $CD69^+CD49a$+ p=0.003, p=0.04, p=0.016, p=0.04; Vγ9Vδ2 $CD69^+CD103^+$ p=0.005, p=0.0009; Vγ9Vδ2 $CD69^+CD49a^+$ p=0.003, p=0.0005, p=0.04). **h,** Representative flow cytometry plot and summary data of IFNγ and TNFα expression by ZOL/IL-2 expanded Vγ9Vδ2 T-cells unstimulated, directly treated with ZOL 5μM, or after co-culture with untreated or ZOL pre-treated HuH7 cells; all conditions performed in triplicate (n=10; IFN-γ p=0.0003, p=0.008, p=0.01; TNF-α p=0.0001, p=0.002). **i,** IFN-γ and TNF-α expression by ZOL/IL-2 expanded Vγ9Vδ2 T-cells after co-culture with ZOL pre-treated HuH7 cells, with or without Mevastatin 100µM or 200µM pre-treatment (n=10; IFN-γ p=0.002; TNF-α p=0.0002, p=0.049). Two-tailed p-values were determined using Friedman test with Dunn's post-hoc test for multiple comparisons **(a,d-f,g-i)**, Wilcoxon matched-pairs signed rank test **(b,d),** Mann-Whitney U test **(c)**. Error bars, mean ± SEM. * p<0.05; ** p<0.01; *** p<0.001.

Supp.Table.1

Clinical details of patients who received a donor and recipient HLA-mismatched liver transplant (n=2).

	Recipient HLA status	Donor HLA status	Year of original OLT	Time to re-transplant	Indication for original OLT	Indication for re-OLT
1	A01<br>A03<br>B07<br>B47	A02<br>A03<br>B07	2008	11 yrs	Drug-induced liver failure	Biliary complications with allograft cirrhosis
2	A01<br>A31<br>B40	A02<br>A03<br>B08	2012	6yrs 8 months	ALD/HCV-related HCC	Hepatic artery thrombosis (with multiple biliary abscesses)

OLT, orthotopic liver transplant; ALD, alcohol-related liver disease; HCV, Hepatitis C virus; HCC, hepatocellular carcinoma.

Supp.Table.2

Clinical details of patients with HCC who underwent curative surgical resection (n=28), with immunohistochemistry performed on paired HCC and background liver samples.

Patient demographics	N=28
Sex	
Male	21
Female	7
Median age at diagnosis (range)	66 (30-89)
Underlying liver disease	
Cirrhosis	10
Fibrosis	2
Steatosis	2
No underlying liver disease	14
Aetiology of Chronic Liver Disease (if present)	
HCV	5
HBV	4
ALD	3
NASH	5
Other	2
If cirrhotic (n=10), Child Pugh Grade	
A	9
B	1
Median diameter largest liver lesion (range) (cm)	5.25 (1.9-15.6)
Number of liver lesions	
Single	25
≥2	3
Histological grade of HCC	
Well differentiated	5
Moderately differentiated	20
Poorly differentiated	3
First onset of HCC	
Yes	25
No (recurrence from previous HCC)	3
Recurrence of HCC post procedure	
Yes	11
No	17
3-year overall survival	
Yes	22
No	5
Unknown	1

HCV, Hepatitis C virus; HBV, Hepatitis B virus; ALD, alcohol-related liver disease; NASH, non-alcoholic steatohepatitis; HCC, hepatocellular carcinoma.

Supp.Table.3

Clinical details of patients with HCC who underwent curative surgical resection or liver transplantation included in flow cytometry analysis (n=27).

**Patient demographics**	**N=27**
Sex	
Male	23
Female	4
Median age at procedure (range)	64 (40-76)
Surgical procedure	
Resection	17
Transplant	10
Cirrhosis	
Yes	21
No	6
Aetiology of Chronic Liver Disease (if present)	
HCV	9
HBV	1
ALD	6
NASH	7
Other	1
MELD score: median (range)	9 (6-18)
If cirrhosis present (n=19), Child Pugh Grade	
A	14
B	7
Median diameter of largest liver lesion (range) (cm)	5 (1.1-13.4)
Number of liver lesions	
Single	18
2-4	7
Multiple satellite lesions	2
Alpha-fetoprotein: median (range)	3.7 (1.4 – 286)
Vascular invasion	
Macrovascular	4
Microvascular	9
None	14
Recurrence of HCC post procedure	
Yes	3
No	24
Survival at last follow-up	
Yes	26
No	1

HCV, Hepatitis C virus; HBV, Hepatitis B virus; ALD, alcohol-related liver disease; NASH, non-alcoholic steatohepatitis; MELD, model for end-stage liver disease; HCC, hepatocellular carcinoma.

Supp.Table.4

Clinical details of patients with CRCLM who underwent curative surgical resection included in flow cytometry analysis (n=33).

**Patient demographics**	**N=33**
Sex	
Male	17
Female	16
Median age at procedure (range)	58 (32 – 82)
Background liver disease	
Yes	1
No	32
Median diameter of largest liver lesion (range) (cm)	4 (0.6 – 11)
Number of liver lesions	
Single	14
2-3	15
≥4	4
Macrovascular invasion	
Yes	6
No	27
Survival at last follow-up	
Yes	29
No	4

CRCLM, Colorectal cancer liver metastases.

Supp.Table.5

Details of monoclonal antibodies used for flow cytometric analysis.

ANTIGEN	FLUOROCHROME	CLONE	SUPPLIER	DILUTION
**CD45**	BUV805	HI30	BD Bioscience (612891)	1:100
**CD3**	APC/Cyanine7	OKT3	Biolegend<br>(317342)	1:100
**CD3**	BUV395	UCHT1	BD Bioscience<br>(563546)	1:100
**TCR pan γδ**	PC7	IMMU510	Beckman Coulter (B10247)	2:100
**TCR pan γδ**	BV421	11F2	BD Bioscience<br>(744870)	2:100
**TCR Vδ1**	FITC<br>Or PE	REA173	Miltenyi<br>(130-118-362/130-120-440)	2:100
**TCR Vδ2**	PE	123R3	Miltenyi<br>(130-095-796)	2:100
**TCR Vδ2**	PE/Cyanine7 or BV605	B6	Biolegend (331422/331430)	2:100
**TCR Vγ9**	APC	B3	Biolegend<br>(331310)	2:100
**CD8a**	BV785	RPA-T8	Biolegend (3010460)	1;100
**CD4**	APC-Cyanine7	RPA-74	BD Bioscience<br>(557871)	1:100
**CD69**	BV605<br>Or PE/Dazzle594	FN50	Biolegend (310938/310942)	1:100
**CD103**	BV711	Ber-ACT8	Biolegend (350222)	2:100
**CD49a**	BUV395 or APC	SR84 or TS217	BD Bioscience (742363)<br>Biolegend 328314	2:100
**CXCR6**	PE/Dazzle594 or BV421	K04IES	Biolegend (356016/356014)	3:100
**CXCR3 (CD186)**	PE	G025H7	Biolegend (353706)	2:100
**HLA-DR**	BV510	G46-6	BD Bioscience (563083)	1:100
**HLA-DR**	eFluor450	L243	eBioscience<br>(48-9952-42)	1:100
**CD27**	BV500	MT271	BD Bioscience (561222)	2:100
**CD45RA**	AlexaFluor700	HI100	Biolegend (304120)	2:100
**CD62L**	PE/Cyanine7	DREG-56	Biolegend (304821)	2:100
**IFN-γ**	BV450	B27	BD Bioscience (560371)	2:100
**TNF-α**	PE/Cyanine7<br>Or AlexaFluor700	MAb11	Biolegend<br>(502930/5029228)	2:100
**IL-2**	PE/Dazzle594	MQ1-17HI2	Biolegend (500344)	2:100
**Granzyme B**	AlexaFluor700	GB11	BD Bioscience (560213)	1:100
**PD-1**	PE	EH12.2H7	Biolegend (329906)	2:100
**HLA-A2**	PE/Cyanine7	BB7.2	Biolegend (343314)	3:100
**CX3CR1**	PE/Dazzle594	2A9-1	Biolegend (341623)	2:100
**Blimp-1**	PE	6D3	BD Bioscieince (564702)	1:100
**T-bet**	BV421	4B10	Biolegend (644816)	2:100
**Eomes**	PE/Dazzle594	WD192B	eBioscience (61487742)	2:100
**Tcf-1**	PE	7F11A10	Biolegend (655207)	2:100
**PD-L1 blocking antibody (anti-CD274)**	Unlabelled	B7-H1	eBioscience (16-5983-82)	2.5μg or 5μg/ml
**TCR pan γδ (plated TCR stim)**	PE/Cyanine7	B1	Biolegend (331222)	4μg/ml
