# Supplementary material for: Characterisation and induction of tissue-resident gamma delta T-cells to target hepatocellular carcinoma
Source: Nat Commun. 2022 Mar 16;13:1372. doi: 10.1038/s41467-022-29012-1 (PMC8927126; doi:10.1038/s41467-022-29012-1)
Supplement: Supplementary file 3 — Reporting Summary [file 41467_2022_29012_MOESM3_ESM.pdf]

nature research

Corresponding author(s): Maini MK

Last updated by author(s): 16/1/2022

Reporting Summary

Nature Research wishes to improve the reproducibility of the work that we publish. This form provides structure for consistency and transparency in reporting. For further information on Nature Research policies, see Authors & Referees and the Editorial Policy Checklist.

Statistics

For all statistical analyses, confirm that the following items are present in the figure legend, table legend, main text, or Methods section.

n/a	Confirmed	
☐	☒	The exact sample size (*n*) for each experimental group/condition, given as a discrete number and unit of measurement
☐	☒	A statement on whether measurements were taken from distinct samples or whether the same sample was measured repeatedly
☐	☒	The statistical test(s) used AND whether they are one- or two-sided<br>*Only common tests should be described solely by name; describe more complex techniques in the Methods section.*
☒	☐	A description of all covariates tested
☐	☒	A description of any assumptions or corrections, such as tests of normality and adjustment for multiple comparisons
☐	☒	A full description of the statistical parameters including central tendency (e.g. means) or other basic estimates (e.g. regression coefficient) AND variation (e.g. standard deviation) or associated estimates of uncertainty (e.g. confidence intervals)
☐	☒	For null hypothesis testing, the test statistic (e.g. *F*, *t*, *r*) with confidence intervals, effect sizes, degrees of freedom and *P* value noted<br>*Give P values as exact values whenever suitable.*
☒	☐	For Bayesian analysis, information on the choice of priors and Markov chain Monte Carlo settings
☒	☐	For hierarchical and complex designs, identification of the appropriate level for tests and full reporting of outcomes
☐	☒	Estimates of effect sizes (e.g. Cohen's *d*, Pearson's *r*), indicating how they were calculated

Our web collection on statistics for biologists contains articles on many of the points above.

Software and code

Policy information about availability of computer code

Data collection	Software used for data acquisition:FACSDIVA v8.0 (BD biosciences), Akoya Mantra 2 microscope.
Data analysis	Software used for data/statistical analysis: FlowJo v.9; Prism 7.0; Excel v.16.57, Inform automated image analysis software (Akoya), Gene Expression Profiling Interactive Analysis 2 (GEPIA2) web server (http://gepia2.cancer-pku.cn/#survival).

For manuscripts utilizing custom algorithms or software that are central to the research but not yet described in published literature, software must be made available to editors/reviewers. We strongly encourage code deposition in a community repository (e.g. GitHub). See the Nature Research guidelines for submitting code & software for further information.

Data

Policy information about availability of data

All manuscripts must include a data availability statement. This statement should provide the following information, where applicable:
- Accession codes, unique identifiers, or web links for publicly available datasets
- A list of figures that have associated raw data
- A description of any restrictions on data availability

The raw data underlying all Figures and Supplementary Figures in this study are provided in the Source Data file. The gene expression analysis data obtained from the Cancer Genome Atlas database are publicly available through the Gene Expression Profiling Interactive Analysis 2 (GEPIA2) web server, http://gepia2.cancer-pku.cn/#survival.

Field-specific reporting

Please select the one below that is the best fit for your research. If you are not sure, read the appropriate sections before making your selection.

☒ Life sciences ☐ Behavioural & social sciences ☐ Ecological, evolutionary & environmental sciences

For a reference copy of the document with all sections, see nature.com/documents/nr-reporting-summary-flat.pdf

Life sciences study design

All studies must disclose on these points even when the disclosure is negative.

Sample size	No statistical methods were used to predetermine sample size. The sample size was selected based on similar published studies in the field (Hunter et al. J.Hepatology 2018, Pallett et al. J Exp Med 2017, Wu et al. Sci Trans Med 2019) and on available patient tissue samples.
Data exclusions	All tissue samples with sufficient numbers of isolated viable lymphocytes were included in this study.
Replication	For phenotypic and functional assessment of human T-cells by flow cytometry, experiments were performed with samples from multiple independent donors (n numbers are stated in figure legends). Experimental variation between donors has been depicted graphically with all data points displayed in each figure. The experimental techniques used (e.g. leukocyte isolation, monoclonal antibody staining, flow cytometry, data analysis) were repeatedly performed on independent days. Experiments involving cell co-culture with HepG2 and HuH7 cells were all performed in duplicates or triplicates with several independent donors as stated in the figure legends.
Randomization	No randomization was performed as this was not relevant for the study. All tissue samples with sufficient numbers of isolated viable lymphocytes were included and analysed; fresh tissue samples were used for co-culture experiments according to tissue availability. All tissue donors were pseudonymised and investigators were blinded to clinical data during analysis. Clinical patient data were collected following completion of all experiments and analysis, and correlations were subsequently examined between clinical characteristics and the study findings, as described within the manuscript.
Blinding	During the analysis investigators were blinded to all clinical patient data. During data collection, investigators were only aware of the tissue or tumour-type in order to facilitate correct tissue retrieval and processing but were blinded to all patient clinical data.

Reporting for specific materials, systems and methods

We require information from authors about some types of materials, experimental systems and methods used in many studies. Here, indicate whether each material, system or method listed is relevant to your study. If you are not sure if a list item applies to your research, read the appropriate section before selecting a response.

Materials & experimental systems

n/a	Involved in the study	
☐	☒	Antibodies
☐	☒	Eukaryotic cell lines
☒	☐	Palaeontology
☒	☐	Animals and other organisms
☐	☒	Human research participants
☒	☐	Clinical data

Methods

n/a	Involved in the study	
☒	☐	ChIP-seq
☐	☒	Flow cytometry
☒	☐	MRI-based neuroimaging

Antibodies

Antibodies used	Detailed information regarding the fluorochrome, clone, supplier and catalogue numbers for all antibodies used in this study are listed in Supp.Table5. Antibodies used in the study included: CD45 H30 clone (Cat 612891 BD Bioscience), CD3 OKT3 clone (Cat 317342 Biolegend), CD3 UCHT1 clone (Cat 563546, BD Bioscience), TCR-gamma delta IMMU510 clone (Cat B10247, Beckman Coulter), TCR-gamma delta 11F2 clone (Cat 744870, BD Bioscience), TCR V delta1 REA173 clone (Cat 130-118-362, Miltenyi), TCR V delta2 123R3 clone (Cat 130-095-796, Milentyi), TCR V deta2 B6 clone (Cat 331422, Biolegend), CD8a RPA-T8 clone (Cat 3010460, Biolegend), CD4 RPA-74 clone (Cat 557871, BD Bioscience), CD69 FN50 clone (Cat 310938, Biolegend), CD103 Ber-ACT8 clone (Cat 350222, Biolegend), CD49a SR84 clone (Cat 742363, BD Bioscience), CXCR6 K04IES clone (Cat 356016, Biolegend), CXCR3 G025H7 clone (Cat 353706, Biolegend), HLA-DR G46-6 clone (Cat 563083, BD Bioscience), CD27 MT271 clone (Cat 561222, BD Bioscience), CD45RA HI100 clone (Cat 304120, Biolegend), CD62L DREG-56 (Cat 304821, Biolegend), IFN-gamma B27 clone (Cat 560371, BD Bioscience), TNF-alpha MAb11 clone (Cat 502930, Biolegend), IL-2 MQ1-17HI2 (Cat 500344, Biolegend), Granzyme B GB11 (Cat 560213, BD Bioscience), PD-1 EH12.2H7 clone (Cat 329906, Biolegend), HLA-A2 BB7.2 clone (Cat 343314, Biolegend), CX3CR1 2A9-1 clone (Cat 341623, Biolegend), Blimp-1 6D3 clone (Cat 564702, BD Bioscience), T-bet 4B10 (Cat 644816, Biolegend), Eomes WD192B clone (Cat 61487742, eBioscience), Tcf-1 7F11A10 clone (Cat 655207, eBioscience).
Validation	All antibodies were purchased from well established manufacturers and were validated by the suppliers for species and target,

the validation information can be found on the company's websites.
Clones and catalogue numbers for each antibody have been included for cross-referencing of manufacturing company specification/validation processes in Supp.Table5.
We further validated antibodies by using negative as well as positive controls where possible (using cell populations known to lack or express a certain marker), and by antibody titration to achieve optimal concentrations with minimal background staining, as well as using isotype controls and fluorescence minus one (FMO) controls for accurate data analysis.
Antibody concentrations are stated in Supp.Table5.

Eukaryotic cell lines

Policy information about cell lines

Cell line source(s)	HepG2: purchased from ATCC (Hep G2 [HEPG2] (ATCC® HB-8065™))<br>HuH7: original commercial source unknown, provided in kind by N.Ramamurthy and P.Klenerman, University of Oxford.
Authentication	HepG2 cell lines were commercially purchased from a well established company and were validated by the commercial provider. HuH7 expression confirmed by N.Ramamurthy and our lab. Further HepG2 and HuH7 cell line validation confirmed in-house using flow cytometric analysis for prototypic hepatoma cell line phenotypic markers.
Mycoplasma contamination	All cell lines used in this study are regularly tested negative for mycoplasma contamination by PCR (EZ-PCR Mycoplasm Kit, Biological Industries).
Commonly misidentified lines (See ICLAC register)	No commonly misidentified cell lines were used in this study.

Human research participants

Policy information about studies involving human research participants

Population characteristics	Each study participant provided written informed consent prior to inclusion in the study, with the storage of any samples/clinical information complying with the requirements of the Human Tissue Act 2004 and the Data Protection Act 1998. Peripheral blood samples were taken from healthy control individuals, patients with CRCLM and HCC, and cirrhotic patients without HCC. Tumour-free liver tissue, tumoural tissue and paired blood samples from patients with HCC or CRCLM undergoing surgical resection or liver transplantation, were obtained through the Tissue Access for Patient Benefit (TapB) scheme at The Royal Free Hospital NHS Foundation Trust. Clinical details of all patients are provided in Supp.Tables 1-4. Covariate-relevant population characteristics such as age and gender and background liver disease were recorded for each patient cohort (Supp.Tables 1-4) and analysed for correlations with study findings, as described within the manuscript. The patient population with HCC consisted of a greater proportion of male participants, which was expected given a well-recognised higher prevalence of HCC in this patient subgroup.
Recruitment	Peripheral blood samples were taken from healthy control individuals (healthy volunteers recruited at UCL), and patients with CRCLM or HCC (recruited at the Royal Free Hospital, London). Resected liver tissue, tumour tissue and paired blood samples were obtained through the Tissue Access for Patient Benefit (TapB) scheme at The Royal Free Hospital. Each participant provided written informed consent before inclusion.<br>Patients were recruited by healthcare professionals independent from the study investigators, and samples were obtained without bias according to tissue accessibility from surgical resection lists or liver transplantation procedures.
Ethics oversight	This study was approved by the local ethics board Brighton and Sussex (Research Ethics Committee reference number 11/LO/0421) and by the University College London–Royal Free Hospital BioBank Ethical Review Committee; Research Ethics Committee reference number 11/WA/0077 and 07/Q0501/50.

Note that full information on the approval of the study protocol must also be provided in the manuscript.

Flow Cytometry

Plots

Confirm that:

☒ The axis labels state the marker and fluorochrome used (e.g. CD4-FITC).

☒ The axis scales are clearly visible. Include numbers along axes only for bottom left plot of group (a 'group' is an analysis of identical markers).

☒ All plots are contour plots with outliers or pseudocolor plots.

☒ A numerical value for number of cells or percentage (with statistics) is provided.

Methodology

Sample preparation	PBMC were isolated from heparinized blood by density centrifugation with Ficoll-Hypaque Plus (GE Healthcare) or Pancoll (Pan Biotech). PBMC were used fresh or were cryopreserved in 10% DMSO (Sigma-Aldrich) prior to further use.<br><br>To isolate IHL and TIL, liver/tumour tissue was cut into small pieces and incubated for 30min at 37C in 0.01% collagenase IV (Invitrogen) and 0.001% DNAse I (Sigma-Aldrich), followed by further mechanical disruption via GentleMACS (Miltenyi Biotech), filtration through a 70um cell strainer (BD Bioscience), removal of parenchymal cells on a 30% Percoll gradient (GE Healthcare)

	and lymphocyte isolation via density centrifugation as described above. IHL and TIL were used immediately after isolation.
Instrument	BD Bioscience Fortessa-X20
Software	FlowJo v.9 (TreeStar/BD Bioscience)
Cell population abundance	Sample purity, where appropriate, was confirmed by flow cytometry to confirm purities >98%.
Gating strategy	The gating strategy used for gamma delta T cell identification in PBMC, IHL and TIL is shown in Supp. Fig1a: cells were gated on their size and granularity to identify lymphocytes, single cells, live cells, CD45+CD3+ T cells, gamma-delta T cells, Vdelta1 and Vdelta2 cells +/- V gamma 9 cells.

☒ Tick this box to confirm that a figure exemplifying the gating strategy is provided in the Supplementary Information.
